# Questionnaire choice affects the prevalence of recommended physical activity: an online survey comparing four measuring instruments within the same sample

**DOI:** 10.1186/s12889-020-10113-9

**Published:** 2021-01-07

**Authors:** Gerrit Stassen, Kevin Rudolf, Madeleine Gernert, Ansgar Thiel, Andrea Schaller

**Affiliations:** 1 Working Group Physical Activity-Related Prevention Research, Institute of Movement Therapy and Movement-Oriented Prevention and Rehabilitation, German Sport University Cologne, Am Sportpark Müngersdorf 6, 50933 Cologne, Germany; 2 Department of Movement-Oriented Prevention and Rehabilitation Sciences, Institute of Movement Therapy and Movement-Oriented Prevention and Rehabilitation, German Sport University Cologne, Am Sportpark Müngersdorf 6, 50933 Cologne, Germany; 3 Institute of Sports Science, Eberhard Karls University Tübingen, Wilhelmstraße 124, 72074 Tübingen, Germany; 4 Interfaculty Research Institute for Sport and Physical Activity, Eberhard Karls University Tübingen, Wilhelmstraße 124, 72074 Tübingen, Germany

**Keywords:** Physical activity, Recommendations, Surveillance, Assessment, Methodology, Epidemiology

## Abstract

**Background:**

Since prevalence estimates of recommended physical activity (PA) considerably vary between different surveys, prevalence might be crucially affected by the choice of measuring instrument. The aim of the present study is to compare the results of four PA questionnaires regarding the current moderate- and vigorous-intensity aerobic PA (MVPA) recommendations of the World Health Organization.

**Methods:**

Within an online survey, participants answered the German Health Interview and Examination Survey for Adults (*DEGS*), the European Health Interview Survey PA Questionnaire (*EHIS*), the Eurobarometer (*EURO*), and a single-item measure (*SIM*). Weekly volume of MVPA was compared via a Friedman test and the prevalence of participants achieving the WHO’s MVPA recommendation via Cochran’s Q. Agreement between all questionnaire pairs was evaluated via Kappa statistics.

**Results:**

One hundred seventy-six participants were included in the analyses (70.5% female, mean age: 33.1 years (SD=12.2)). Between the four questionnaires, the weekly volume of MVPA statistically significant differed (*SIM*: MED=90.0 (MIN=0.0, MAX=210.0), *DEGS*: MED=120.0 (MIN=0.0, MAX=420.0), *EHIS*: MED=24.0 (MIN=0.0, MAX=1395.0), *EURO*: MED=51.0 (MIN=0.0, MAX=2430.0), *p*<.001, all pairwise comparisons *p*<.01), as well as the prevalence of participants achieving the MVPA recommendations (*SIM* 31.3% (95% CI 24.5–38.7)*, DEGS* 43.2% (95% CI 35.8–50.8), *EHIS* 67.0% (95% CI 59.6–73.9), *EURO* 87.5% (95% CI 81.7–92.0), *p*<.001), except between *SIM* and *DEGS* (*p*=.067). Agreement was weak between all questionnaire pairs (all κ< 0.60).

**Conclusions:**

Questionnaire choice crucially affects the resulting MVPA data and hence the prevalence of achieving recommended PA levels within the same sample. Therefore, for PA surveillance, standardised survey and analysis methods and efforts to harmonise monitoring systems are needed, since whether recommended levels of PA are achieved should not be determined by the choice of one measuring instrument or another.

## Background

The multiple positive effects of physical activity (PA) on health are well established [1–4]. At the same time, inactivity has a major negative health effect worldwide [5] and insufficient PA is one of the leading risk factors of global mortality [6], as well as being responsible for a substantial economic burden [7], thus underlining the importance of population-wide PA surveillance based on accurately-collected data.

The global recommendations on PA for health of the World Health Organization (WHO) for adults aged 18–64 years comprise aerobic PA for at least 150 min at moderate intensity or at least 75 min at vigorous intensity throughout the week, or an equivalent combination of moderate- and vigorous-intensity activity (MVPA) [8, 9]. In addition, muscle-strengthening activities and exercises (MSE) involving major muscle groups should be performed on two or more days a week. Comparable recommendations can be found in several national guidelines, e.g. for the United Kingdom [10], the United States [11], Australia [12], and Germany [13].

Worldwide, it has been estimated in recent years that about 30% of adults are physically inactive [14, 15] and the current target of the WHO is a 15% relative reduction in the global prevalence of insufficient PA by 2030 [16]. However, cross-country comparisons show large differences in the percentage of people achieving the recommendations for PA. Reviews report prevalence estimates ranging from 7% up to 96% [17, 18]. Within a single country, Macniven et al. report prevalence ranging from 18 to 92% depending on the respective survey [18].

Comparable ranges can also be found in representative German surveys. In two surveys by the Robert Koch Institute (RKI), prevalence of 20% [19] (survey period: November 2008–December 2011, *n*=7988) and 45% [20] (survey period: November 2014 – July 2015, *n*=22,959) is reported. Another nationwide study again reports a prevalence of 53% [21] (survey period: March – April 2012, *n*=2248), while in the Eurobarometer survey 84% of the German sub-sample reaches the MVPA recommendations [22] (survey period: November – December 2013). Since the studies listed above state that the samples were weighted according to the structure of the German population, and given that they all refer to current recommendations regarding the prevalence of recommended MVPA, it could be assumed that the considerable differences in prevalence estimates may be due to variations in the measurement and analysis of the recommended MVPA, and not necessarily differing PA behaviour among the populations surveyed. The studies used different self-report measures, which raises the question of how the choice of questionnaire alone could affect the prevalence of recommended MVPA.

Already two decades ago, Sarkin et al. [23] examined the results of three questionnaires in terms of achieving two PA guidelines, finding that within the same sample at the same measurement point, the proportion of those meeting the 1990 American College of Sports Medicine (ACSM) fitness guidelines [24] was 32–59% and the proportion of those meeting the 1995 Centers for Disease Control and Prevention (CDC)/ACSM health-related guidelines [25] was 4–70%, respectively, depending on the chosen questionnaire. In a study by Steene-Johannessen et al. [26], similarly wide ranges can be observed in a comparison of three questionnaires within the same sample, as the prevalence of recommended MVPA (≥150 min/week) was between 40 and 88%.

In order to compare prevalence studies conducted with self-report measures regarding current PA recommendations, it is essential that the prevalence is unaffected by the choice of the instrument. However, if the prevalence of recommended PA depends on the chosen questionnaire, this would call into question PA surveillance and the comparability of surveys.

But since it does not seem certain that different questionnaires measuring the same behaviour necessarily lead to the same or comparable prevalence, the present study aims to compare the results of four PA questionnaires, that were recently used in national or international surveys and/or measure recommended PA with a minimum number of items, within one sample in terms of achieving the WHO’s current MVPA recommendations for adults.

## Methods

### Study design

The online survey was created via the software EFS Survey (Questback GmbH, Cologne, Germany) and pilot tested prior to its dissemination. The survey period lasted for 1 month (31st October – 30th November 2019). The study sample was recruited via the website, the official Facebook account, and the official Twitter account of the German Sport University Cologne, as wells as SurveyCircle, a web portal for the acquisition of study participants, and associated Twitter accounts. Participants were invited to answer an online survey regarding the measurement of PA and the comparison of questionnaires. Prior to the start of the survey, participants were informed about anonymity and that the data would be evaluated for scientific purposes. Participants received no financial incentives.

### Measures

During the online survey, each participant answered four PA questionnaires (German versions) in one session one after the other, with the survey software randomising the order to balance order effects:

- the PA-related questions of the German Health Interview and Examination Survey for Adults (DEGS1) [19, 27] (*DEGS*);
- the European Health Interview Survey-Physical Activity Questionnaire [28] (*EHIS*);
- the PA-related questions of the Eurobarometer survey, wave 80.2 [29] (*EURO*); and
- a single-item measure (*SIM*) (self-translation of a PA screening tool by Milton et al. [30]).

All questionnaires have recently been used in population-wide surveys and/or – according to corresponding publications – in case of the *SIM* provide a short self-report option to determine whether the respondents achieve current MVPA recommendations (≥150 min/week) (Table 1).

**Table 1:** Description of the included measuring instruments, the calculations of the weekly aerobic physical activity, and the comparison with the recommended benchmark

#	Questionnaire	Abbr. used	Number of items	Recall period	Description of questions	Answer options	Intensity description	Minimum bout length	Included domains for the MVPA recommendations comparison	Calculation of MVPA and comparison with the benchmark (≥150 min/week)
1	German Health Interview and Examination Survey for Adults	*DEGS*	2	Typical week in the last 3 months	1: number of days with PA; 2: average duration of PA	1: number or on no day; 2: less than 10 min, 10 to less than 30 min, 30 to less than 60 min, more than 60 min	Physically active in a way that one sweats or gets out of breath	Not named	Not specified	Number of days is multiplied by the mean of the response category (the top category is estimated at 60 min) and compared to the benchmark
2	European Health Interview Survey - Physical Activity Questionnaire	*EHIS*	8	Typical week	(Conservatively, only items 4–7 are used for MVPA calculation if walking is not included) 1: description of main work; 2: number of days with walking (to get to and from places); 3: average duration of walking; 4: number of days with cycling (to get to and from places); 5: average duration of cycling; 6: number of days with leisure-time/recreational PA (e.g. brisk walking, ball games, jogging, cycling, swimming) 7: total time of leisure-time/recreational PA 8: number of days with muscle-strengthening activities and exercises	4: number (1–7) or on no day; 5: 10–29 min, 30–59 min, 1 h to less than 2 h, 2 h to less than 3 h, 3 h or more 6: number (1–7) or on no day; 7: total time in h and min	Physically active in a way that causes at least a small increase in breathing or heart rate (for leisure-time/recreation)	Only for cycling (at least 10 min)	Leisure-time/recreation, transportation	The number of days (cycling) is multiplied by the mean of the response category and added to total weekly leisure-time PA before the sum is then compared to the benchmark
3	Eurobarometer	*EURO*	6	Last 7 days	1: vigorous PA (e.g. lifting heavy things, digging, aerobics, fast cycling); 3: moderate PA (e.g. carrying light loads, cycling at normal pace, doubles tennis); 5: walking; 2, 4, and 6: Average duration of PA	1, 3, and 5: number of days (0–7); 2, 4, and 6: 30 min or less, 31 to 60 min, 61 to 90 min, 91 to 120 min, more than 120 min, never do [type of activity]	Description is indirect via the examples in the questions	Only for walking (at least 10 min)	Leisure-time/recreation, transportation, occupational/household (via examples)	Number of days for each activity is multiplied by the mean of the response category (the top category is estimated at 120 min) and that for vigorous activity is doubled before all is summed up and then compared to the benchmark
4	Single-item measure	*SIM*	1	Past week	1: number of days with at least 30 min PA (include: sport, exercise, brisk walking or cycling for recreation or transport, exclude: household- or job-related)	1: number of days (0–7)	Physically active in a way that increases the breathing rate	Not named	Leisure-time/recreation, transportation	Value of ≥5 days means that the benchmark has been achieved

The *DEGS* comprises two items. The first asks about the number of days in an average week during the last 3 months with PA on which the respondent had started sweating or found themselves of breath [19]. The wording was based on the CDC recommendations [25] to raise the respondents’ awareness of the recommended minimum intensity. The second item asks about the daily duration of PA on each of these days with the following options: less than 10 min, 10 to less than 30 min, 30 to less than 60 min, more than 60 min. In the corresponding RKI publication, no information on reliability and validity is given, but “it is possible to approximately estimate the proportion of those who fulfil the WHO recommendation of 2.5h/week.” [19]. Permission for the scientific use of this measuring instrument was obtained in advance from the RKI.

The *EHIS* comprises eight items regarding PA in different domains (workplace, transport, leisure time, muscle-strengthening) during a typical week “and [it] allows to estimate the health-enhancing PA recommendation compliance” [28]. First, a description of the time spent on work is asked for. The next four items (transport) ask about the number of days per week on which the respondet walked or cycled for at least 10 min and the respective duration (response options: “10-29 minutes per day”, “30-59 minutes per day”, “1 hour to less than 2 hours per day”, “2 hours to less than 3 hours per day”, “3 hours or more per day”). The next two items ask first about the number of days per week with at least 10 min of sports, fitness or recreational (leisure) PA (excluding work and transport), showing at least a slight increase in breathing or heart rate, and second about the total weekly time (being expressed in hours and minutes per week). Test-retest reliability for aerobic health-enhancing PA has a correlation coefficient of 0.43 and concurrent validity coefficients with self-report and objective criterion measures are 0.41–0.64 [31]. In addition. The final item asks about the number of days with activities specifically designed to strengthen muscles (resistance training or strength exercises) [28], whereby it is the only one among the questionnaires included in the present study to survey the whole WHO PA recommendations. The measuring instrument was taken from the questionnaire of the German Health Update 2014/2015 – European Health Interview Survey of the RKI, which may be reused for scientific purposes [32].

The *EURO* comprises six items that ask about PA via the number of days of vigorous activity, moderate activity (excluding walking), and walking for at least 10 min in the last 7 days [29] and the respective daily duration (response options: “30 min or less”, “31 to 60 min”, “61 to 90 min”, “91 to 120 min”, “more than 120 min”, “never do [type of activity]”, “don’t know”) “in order to assess the levels of physical activity ( …) according to the WHO’s recommendations” [22]. The items are slightly modified items of the International Physical Activity Questionnaire [22], which shows acceptable reliability [33, 34] but low concurrent validity in terms of correlations with objective measurement methods [33, 35]. The measuring instrument is freely available online [29].

The *SIM* uses a past-week recall period asking about the number of days with at least 30 min of PA with an intensity that raises the breathing rate, including sport, exercise, walking, and cycling for recreation, but excluding housework and work-related PA. For the present study, the German version [36] of the single-item measure by Milton et al. [30] – whose wording refers to the recommendations of 30 min of moderate intensity activity on five or more days of the week [37] – was slightly rephrased. The underlying measure shows strong reproducibility (test-retest correlation coefficient 0.72) and modest validity regarding the number of days of MVPA against the Global Physical Activity Questionnaire (correlation coefficient 0.53) [30] and accelerometry (0.40–0.54) [36, 38].

Additionally, self-report data on sex, age, height and weight (for body mass index (BMI) calculation), school-leaving and professional qualification to classify the level of education following the international standard [39], and self-perceived health via the first question of the Minimum European Health Module [40] (“How is your health in general?”, response options: “very good”/“good”/“fair”/“bad”/“very bad”, German translation from the German Health Update survey [32]) were asked.

The items of the aforementioned PA questionnaires were mandatory within the online survey to avoid missing values. The additional personal data was voluntary.

### Statistical analyses and visualisation

For the analyses, the sample was limited to the age range of 18–64 years to follow the corresponding target group of the WHO’s PA recommendations for adults [8, 9]. Descriptive analyses were conducted for sex, age, BMI, level of education, and self-perceived health.

For *DEGS*, *EHIS*, *EURO*, and *SIM*, the volume of weekly MVPA was calculated according to the questionnaire-specific calculations (Table 1) and, based on this, participants were classified concerning whether or not they achieved the WHO’s recommendations compared to the benchmark (≥150 min/week).

The weekly volume of MVPA according to the four questionnaires was compared via a Friedman test (Dunn-Bonferroni post-hoc tests). Additionally, to visualise the ranges of weekly MVPA, a radar chart was created. Within the diagram, light grey rectangles illustrate all individual values across the four questionnaires, while two black rectangles show the benchmark and – for comparison – the *DEGS*, *EHIS*, *EURO*, and *SIM* medians.

Cochran’s Q was used to determine whether the proportions of participants achieving the WHO’s MVPA recommendations differend among the questionnaires (Dunn-Bonferroni post-hoc tests).

Levels of agreement between all possible questionnaire pairs regarding achieving the WHO’s MVPA recommendations were evaluated via percent agreement and Kappa statistics with values κ<.60 indicating inadequate or weak agreement for health-related studies, respectively [41].

Statistical significance was set at *p*<.05. All statistical analyses were run with SPSS 27 (IBM Corp., Armonk, NY, USA).

Finally, using the *EHIS* data, participants were additionally classified as achieving or not achieving the WHO’s MSE recommendations and both recommendations combined.

## Results

### Sample description

During the survey period, the questionnaire was completed 180 times (31.6% of 569 accesses). Four records were removed due to an age > 64 years resulting in a total sample of 176 participants (mean age: 33.1 years (SD=12.2). The majority of the participants were female, highly educated, had a good self-perceived health status, and the mean BMI was in the normal range (Table 2).

**Table 2:** Sample characteristics (*n*=176)

Characteristic	
Sex [female] (n (%))	124 (70.5)
Age [years] (mean (SD))	33.1 (12.2)
BMI [kg/m2] (mean (SD))	23.4 (3.9)
Level of education (n (%))	
High	109 (62.3)
Medium	65 (37.1)
Low	1 (0.6)
Health status (5=very good, 1=very bad) (mean (SD))	4.2 (0.7)
Very good/good (n (%))	145 (82.9)
Fair/bad/very bad (n (%))	30 (17.1)

Note: Valid percentages due to missing data

### Questionnaire comparisons

**Fig. 1:**
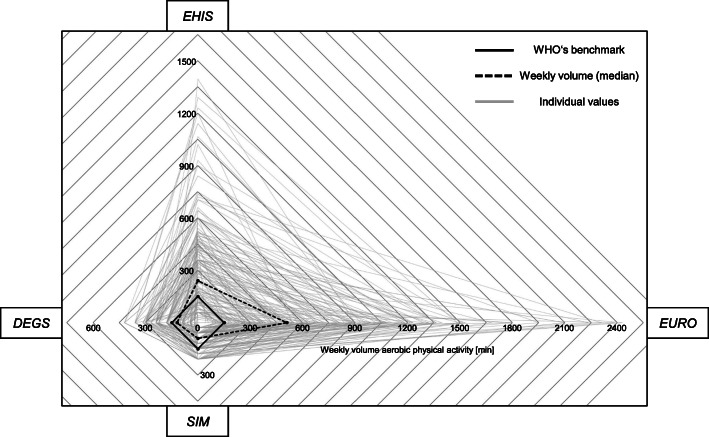
Weekly volume of aerobic physical activity across the four questionnaires

The weekly volume of MVPA was lowest for *SIM* (MED=90.0, MIN=0.0, MAX=210.0) and highest for *EURO* (MED=510.0, MIN=0.0, MAX=2430.0) (*DEGS*: MED=120.0, MIN=0.0, MAX=420.0, *EHIS*: MED=240.0, MIN=0.0, MAX=1395.0). Differences were statistically significant (χ^2^(3)=346.598, *p*<.001) in all pairwise comparisons (pairwise Dunn-Bonferroni post-hoc tests *p*<.01). The radar chart (Fig. 1) visualises the weekly volume of MVPA according to the four questionnaires compared to the benchmark (≥150 min/week). The light grey rectangles illustrate the individual spans of all survey respondents.

**Fig. 2:**
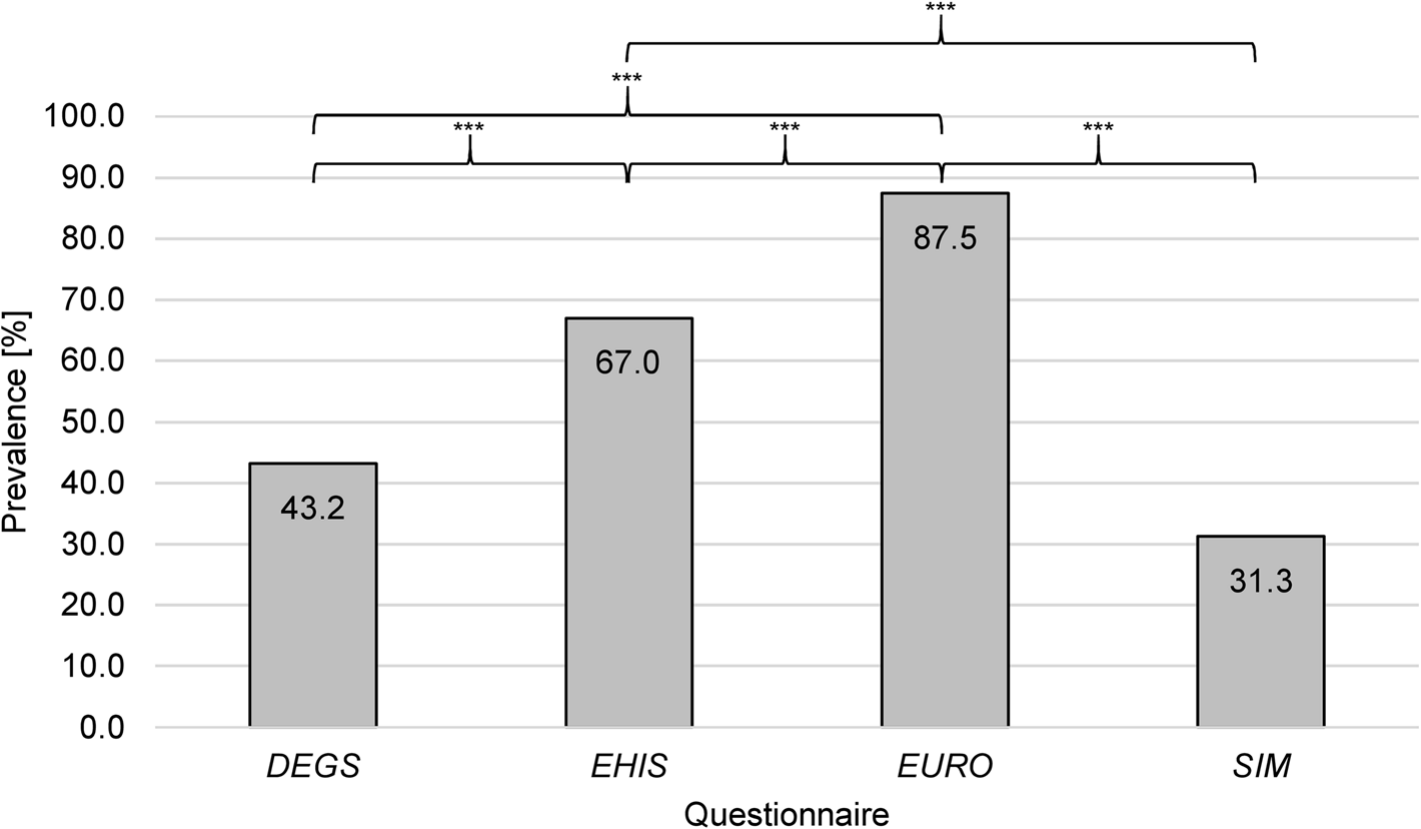
Prevalence of recommended aerobic physical activity across the four questionnaires

The prevalence of participants achieving WHO’s MVPA recommendations ranged from *SIM* 31.3% (95% CI 24.5–38.7) to *EURO* 87.5% (95% CI 81.7–92.0) (*DEGS* 43.2% (95% CI 35.8–50.8), *EHIS* 67.0% (95% CI 59.6–73.9)) (Fig. 2). Cochran’s Q test determined a statistically significant difference in the proportion across the four questionnaires (χ^2^(3)=170.474, *p*<.001). Pairwise Dunn-Bonferroni post-hoc tests were statistically significant between all questionnaires (*p*<.001), except between *SIM* and *DEGS* (*p*=.011, corrected *p*=.067).

Levels of agreement between all possible questionnaire pairs regarding the classification of respondents as achieving or not achieving the MVPA recommendations were weak (all κ< 0.60) (Table 3). The κ-values were between 0.10 for *EURO* and *SIM* (95% CI 0.05–0.16) and 0.41 for *DEGS* and *EHIS* (95% CI 0.30–0.53) and *DEGS* and *SIM* (95% CI 0.28–0.55), respectively.

**Table 3:** Pairwise agreements regarding the number (%) of respondents classified as achieving or not achieving recommended aerobic physical activity

κ=0.41 (95% CI 0.30–0.53)		*EHIS*			κ=0.18 (95% CI 0.09–0.26)		*EURO*			κ=0.41 (95% CI 0.28–0.55)		*SIM*		
		No (%)	Yes (%)	Total (%)			No (%)	Yes (%)	Total (%)			No (%)	Yes (%)	Total (%)
*DEGS*	No (%)	52 (29.5)	48 (27.3)	100 (56.8)	*DEGS*	No (%)	21 (11.9)	79 (44.9)	100 (56.8)	*DEGS*	No (%)	86 (48.9)	14 (8.0)	100 (56.8)
	Yes (%)	6 (3.4)	70 (39.8)	76 (43.2)		Yes (%)	1 (0.6)	75 (42.6)	76 (43.2)		Yes (%)	35 (19.9)	41 (23.3)	76 (43.2)
	Total (%)	58 (33.0)	118 (67.0)	176 (100)		Total (%)	22 (12.5)	154 (87.5)	176 (100)		Total (%)	121 (68.8)	55 (31.3)	176 (100)
κ=0.30 (95% CI 0.16–0.44)		*EURO*			κ=0.18 (95% CI 0.08–0.29)		*SIM*			κ=0.10 (95% CI 0.05–0.16)		*SIM*		
		No (%)	Yes (%)	Total (%)			No (%)	Yes (%)	Total (%)			No (%)	Yes (%)	Total (%)
*EHIS*	No (%)	17 (9.7)	41 (23.3)	58 (33.0)	*EHIS*	No (%)	49 (27.8)	9 (5.1)	58 (33.0)	*EURO*	No (%)	21 (11.9)	1 (0.6)	22 (12.5)
	Yes (%)	5 (2.8)	113 (64.2)	118 (67.0)		Yes (%)	72 (40.9)	46 (26.1)	118 (67.0)		Yes (%)	100 (56.8)	54 (30.7)	154 (87.5)
	Total (%)	22 (12.5)	154 (87.5)	176 (100)		Total (%)	121 (68.8)	55 (31.3)	176 (100)		Total (%)	121 (68.8)	55 (31.3)	176 (100)

Finally, according to the results of the *EHIS* and taking into account the item on MSE, 48.9% of the participants achieved the WHO’s MSE recommendations and 42.0% achieved both recommendations combined.

## Discussion

The present study shows that the questionnaire choice crucially affects the prevalence of recommended PA within the same sample. The prevalence estimates of achieving the WHO’s MVPA recommendations ranged from just over 30% to almost 90% within the same sample and agreement between the questionnaire pairs regarding the classification of respondents (achieving/not achieving MVPA recommendations) was weak.

The results are consistent with previous studies finding that different self-reporting measures within the same sample classify survey participants differently in terms of achieving the recommended PA [23, 26, 42]. For example, the study by Steene-Johannessen et al. [26] also used questionnaires that were employed in large surveys yet found substantial discrepancies in the prevalence estimates [26]. Accordingly, such studies and our results suggest that differences in the prevalence estimates of recommended MVPA between studies which use different questionnaires – e.g. in country comparisons [14, 17, 18] – are not necessarily due to the different PA behaviour of those surveyed, but rather are more likely to be affected by differences in the measuring instruments chosen [43]. In the review by Macniven et al., the prevalence estimates substantially vary – with similar time periods – within eleven of the thirteen countries with more than one survey [18].

It seems obvious that instrument-specific differences in terms of measured activity and the methods used to calculate the achievement of the MVPA recommendations are reasons for the differing prevalence estimates in the present study. Although all included questionnaires could be used to determine the prevalence of recommended MVPA, they more or less substantially differ in terms of intensity descriptions, the activities surveyed, recall periods, and minimum bouts of MVPA. Moreover, the respective calculations of weekly aerobic PA are based on frequencies, total durations or combinations of both. Even if surveys refer to comparable activity recommendations, differences in instrument design seem to lead to different prevalence estimates per se.

Beyond that, it might even be necessary to investigate how collected data should be analysed and to more fundamentally investigate what kind of PA should be measured in order to accurately survey health-enhancing PA. A study by Mealing et al., e.g., has already shown that the use of different scoring algorithms alone (frequency, duration, volume) leads to substantial variations in the estimation regarding the prevalence of recommended MVPA [44]. In its new recommendations for 2020 [9] compared to the recommendations for 2010 [8], the WHO recently removed the requirement of at least 10-min bouts of PA, thus focusing only on the weekly volume. But in addtion, although all domains are essentially mentioned in the WHO’s PA recommendations (leisure-time/recreation, transportation, occupational/household) [8, 9] to reach the recommended weekly volume, there is currently further discussion in research about the extent to which PA is beneficial to health regardless of the setting, or whether a distinction should be made between leisure-time and transport PA and work-related activity [45–48]. It is self-explanatory that the methodological decision to (not) measure certain domain-specific PA has a significant impact on the prevalence of recommended PA [18]. This indicates that questionnaires need to be further adapted to be up to date with the evolving PA recommendations [49] and also with possible new research findings.

Independent of discussions on PA benchmarks for health [45, 50], consideration may need to be given to the standardisation of PA measures in surveillance of the achievement of PA recommendations [14, 17, 18]. Strain et al. concluded in a recent narrative review that due to different PA surveillance measures in the home countries of the United Kingdom, the extent to which PA recommendations are met, currently cannot be compared across the countries [43]. Although there are already efforts to use the same questionnaire in several countries (e.g. World Health Survey [51], International Prevalence Study on Physical Activity [52], or WHO STEPwise approach), the limited comparability of the instruments means that such multi-country data cannot be juxtaposed. Moreover, a convincing argument against methodological standardisation is that it may require the interruption of trend data that is relevant e.g. from a political perspective [43]. However, a study by Carlson et al. showed not only cross-sectional differences between three surveillance systems in the United States, but also slightly different physical activity trends over several years of observation [53], which questions the added value of parallel longitudinal studies carried out with different instruments. Therefore, in order to provide comparable, valid and reliable PA data, harmonised monitoring systems need to be implemented, which is the objective of the EUPASMOS project [54], for example. Another potential approach would be to harmonise data to a compatible format using indirect models (via bridge equations and intermediate values) [55]. The goal of comparable prevalence estimates – regardless of the instrument used and only depending on the PA behaviour of the persons investigated – should be further pursued to create a reliable data basis for PA promotion strategies.

An additional option to reduce uncertainties regarding the comparability of multiple surveys, should be objective measuring instruments. Without question, PA questionnaires are practical and economical for population-based surveys [56, 57], but they still tend to be inaccurate compared to objective instruments, such as accelerometers or pedometers [58–60], and they rarely show good results in terms of both reliability and validity [33, 61]. Moreover, regarding the classification of persons in terms of achieving the MVPA recommendations, self-reports show low or moderate sensitivity compared to objective measurement methods and low levels of agreement [26, 42, 62]. Consequently, the potential and utility of integrating device-based measures into PA surveillance or a combination of objective and subjective measurement methods should be considered to validly and reliably survey the (WHO’s) whole PA recommendations [26, 38, 43, 63, 64].

Notwithstanding the challenges of a comparable MVPA surveillance, another crucial gap remains, as MSE recommendations have only been integrated in a few PA surveillance studies to date [65, 66], which is why Strain et al. accordingly called them “forgotten guidelines” [67]. MSE offer multiple health benefits such as improved physical performance and functional independence, and it assists in the prevention and management of numerous health complaints and diseases [68–71]. Furthermore, scientific findings emphasise the independent positive effects of MSE and that achieving the MSE recommendations appears to be at least as important as adherence to the MVPA recommendations in terms of reducing mortality risk [72, 73]. In addition, the prevalence for achieving both the WHO’s PA recommendations combined seems to be constantly lower than for MVPA alone (some examples: Australia: 53% for MVPA, 19% for MSE, and 15% for both combined [74]; Finland: 31% for MVPA, 17% for MSE, and 11% for both [75]; Germany: 45% for MVPA, 29% for MSE, and 22% for both [20]). Due to MSE’s strong relevance from a public health perspective [70], researchers should therefore choose a survey method that reflects both recommendations and the measurement of MSE should be included in population surveys [43], although MSE can currently be exclusively assessed by self-report and not device-based [65]. Alternatively, study authors should at least distinguish accurately between MVPA and MSE when reporting the prevalence of recommended PA.

The bottom line of the present study is that the measurement of recommended PA strongly depends on the questionnaire itself (the inclusion of different types of PA and the corresponding calculation method). Even if differences in instrument design and analysis methods may be sources of the wide variability, all instruments included in this study indicate that they could be used to survey the prevalence of people achieving the WHO’s MVPA recommendations. However, there is limited interchangeability and prevalence widely varies within the same sample.

### Strengths and limitations

The main strength of the study is that it is a comparison of established PA questionnaires within the same sample with a direct link to the WHO’s PA recommendations, thus providing an important contribution to the discussion on PA surveillance and being highly relevant for future political measures in terms of reducing the prevalence of insufficient PA. Three of the four measuring instruments have already been used in recent years within national and international surveys [19, 20, 22].

For the *DEGS*, no information on reliability and validity could be found from the RKI [19] and for the *SIM,* the German version [36] an established instrument [30] was slighty rephrased. However, the aim of the present study was not to use instruments that are most valid, but rather to compare the influence of the choice of instruments on the prevalence estimates. For this purpose, we used instruments that were frequently used in population-based surveys as well as the SMI, which can very simply survey the achievement of PA recommendations.

Due to the recruitment strategy (online channels of the German Sport University Cologne and publication on SurveyCircle), it is likely that mainly younger, educated and health-oriented people accessed the online survey. However, due to the homogeneity of this positive sample, it could be assumed that the chance of correctly answered questionnaires was increased [76–79]. Future studies with more heterogeneous samples could consider the influence of different variables (e.g. educational level or age) or aim for a comparable measurement method comparison in specific subgroups (e.g. very active athletes or inactive persons).

## Conclusions

At present, data on the prevalence of recommended PA – if collected through a questionnaire – must be viewed sceptically against the background of the measuring instrument used. Our study underlines the need for standardised survey and analysis methods in PA surveillance within and between countries [14, 18, 43], and efforts should be undertaken to harmonise monitoring systems accordingly. Especially at the individual level, the inter-method differences can be very large and the agreement weak, meaning that the results are not necessarily interchangeable [26, 42, 80].

As a solid basis for political measures to reduce physical inactivity, accurately-collected and comparable data is needed and it should not be the choice of one questionnaire or another that determines whether the prevalence of recommended PA is low or high, but rather the actual PA behaviour of those surveyed.

## Data Availability

The datasets used and analysed during the current study are available from the corresponding author on reasonable request.

## Abbreviations

Abbr: Abbreviation
BMI: Body mass index
CDC: Centers for Disease Control and Prevention
CI: Confidence interval
*DEGS*: Physical activity-related questions of the German Health Interview and Examination Survey for Adults
*EHIS*: European Health Interview Survey Physical Activity Questionnaire
*EURO*: Physical activity-related questions of the Eurobarometer survey
h: Hours
MAX: Maximum
MED: Median
MIN: Minimum
min: Minutes
MSE: Muscle-strengthening activities and exercises
MVPA: Moderate- and vigorous-intensity activity
PA: Physical activity
RKI: Robert Koch Institute
SD: Standard deviation
*SIM*: Single-item measure
WHO: World Health Organization
