# Correction to: Questionnaire choice affects the prevalence of recommended physical activity: an online survey comparing four measuring instruments within the same sample

**DOI:** 10.1186/s12889-021-10514-4

**Published:** 2021-04-08

**Authors:** Gerrit Stassen, Kevin Rudolf, Madeleine Gernert, Ansgar Thiel, Andrea Schaller

**Affiliations:** 1 Working Group Physical Activity-Related Prevention Research, Institute of Movement Therapy and Movement-Oriented Prevention and Rehabilitation, German Sport University Cologne, Am Sportpark Müngersdorf 6, 50933 Cologne, Germany; 2 Department of Movement-Oriented Prevention and Rehabilitation Sciences, Institute of Movement Therapy and Movement-Oriented Prevention and Rehabilitation, German Sport University Cologne, Am Sportpark Müngersdorf 6, 50933 Cologne, Germany; 3 Institute of Sports Science, Eberhard Karls University Tübingen, Wilhelmstraße 124, 72074 Tübingen, Germany; 4 Interfaculty Research Institute for Sport and Physical Activity, Eberhard Karls University Tübingen, Wilhelmstraße 124, 72074 Tübingen, Germany

**Correction to: BMC Public Health 21, 95 (2021)**

**https://doi.org/10.1186/s12889-020-10113-9**

It was highlighted that the original article [1] contained some typesetting mistakes. This correction article shows the incorrect and the correct text passages. The Publisher would like to apologize to the authors and readers for any inconvenience caused. The original article has been updated.

Incorrect

Between the four questionnaires, the weekly volume of MVPA statistically significant differed (*SIM*: MED = 90.0 (MIN = 0.0, MAX = 210.0), *DEGS*: MED = 120.0 (MIN = 0.0, MAX = 420.0), *EHIS*: MED = 24.0 (MIN = 0.0, MAX = 1395.0), *EURO*: MED = 51.0 (MIN = 0.0, MAX = 2430.0), *p* < .001, all pairwise comparisons *p* < .01), as well as the prevalence of participants achieving the MVPA recommendations (*SIM* 31.3% (95% CI 24.5–38.7)*, DEGS* 43.2% (95% CI 35.8–50.8), *EHIS* 67.0% (95% CI 59.6–73.9), *EURO* 87.5% (95% CI 81.7–92.0), *p* < .001), except between *SIM* and *DEGS* (*p* = .067).

Correct

Between the four questionnaires, the weekly volume of MVPA statistically significant differed (*SIM*: MED = 90.0 (MIN = 0.0, MAX = 210.0), *DEGS*: MED = 120.0 (MIN = 0.0, MAX = 420.0), *EHIS*: MED = 240.0 (MIN = 0.0, MAX = 1395.0), *EURO*: MED = 510.0 (MIN = 0.0, MAX = 2430.0), *p* < .001, all pairwise comparisons *p* < .01), as well as the prevalence of participants achieving the MVPA recommendations (*SIM* 31.3% (95% CI 24.5–38.7)*, DEGS* 43.2% (95% CI 35.8–50.8), *EHIS* 67.0% (95% CI 59.6–73.9), *EURO* 87.5% (95% CI 81.7–92.0), *p* < .001), except between *SIM* and *DEGS* (*p* = .067).

Incorrect

For example, the study by Steene-Johannessen et al. [26] also used questionnaires that were employed in large surveys yet found substantial discrepancies in the prevalence estimates [26].

Correct

For example, the study by Steene-Johannessen et al. [26] also used questionnaires that were employed in large surveys yet found substantial discrepancies in the prevalence estimates.

Incorrect

Moreover, regarding the classification of persons in terms of achieving the MVPA recommendations, self-reports show low or moderate sensitivity compared to objective measurement methods and low levels of agreement [26, 42, 62]. Consequently, the potential and utility of integrating device-based measures into PA surveillance or a combination of objective and subjective measurement methods should be considered to validly and reliably survey the (WHO’s) whole PA recommendations [26, 38, 43, 63, 64] .

Correct

Moreover, regarding the classification of persons in terms of achieving the MVPA recommendations, self-reports show low or moderate sensitivity compared to objective measurement methods and low levels of agreement [26, 42]. Consequently, the potential and utility of integrating device-based measures into PA surveillance or a combination of objective and subjective measurement methods should be considered to validly and reliably survey the (WHO’s) whole PA recommendations [26, 43, 62–64].

Incorrect

For this purpose, we used instruments that were frequently used in population-based surveys as well as the SMI, which can very simply survey the achievement of PA recommendations.

Correct

For this purpose, we used instruments that were frequently used in population-based surveys as well as the *SIM*, which can very simply survey the achievement of PA recommendations.
